# Low Abdominal NIRS Values and Elevated Plasma Intestinal Fatty Acid-Binding Protein in a Premature Piglet Model of Necrotizing Enterocolitis

**DOI:** 10.1371/journal.pone.0125437

**Published:** 2015-06-10

**Authors:** Irving J. Zamora, Barbara Stoll, Cecilia G. Ethun, Fariha Sheikh, Ling Yu, Douglas G. Burrin, Mary L. Brandt, Oluyinka O. Olutoye

**Affiliations:** 1 Division of Pediatric Surgery, Michael E. DeBakey Department of Surgery, Baylor College of Medicine, Texas Children’s Hospital, Houston, Texas, United States of America; 2 Department of Pediatrics, Baylor College of Medicine, USDA/ARS Children’s Nutrition Research Center, Houston, Texas, United States of America; Vanderbilt University, UNITED STATES

## Abstract

To identify early markers of necrotizing enterocolitis (NEC), we hypothesized that continuous abdominal near-infrared spectroscopy (A-NIRS) measurement of splanchnic tissue oxygen saturation and intermittent plasma intestinal fatty-acid binding protein (*p*I-FABP) measured every 6 hours can detect NEC prior to onset of clinical symptoms. Premature piglets received parenteral nutrition for 48-hours after delivery, followed by enteral feeds every three hours until death or euthanasia at 96-hours. Continuous A-NIRS, systemic oxygen saturation (SpO_2_), and heart rate were measured while monitoring for clinical signs of NEC. Blood samples obtained at 6-hour intervals were used to determine *p*I-FABP levels by ELISA. Piglets were classified as fulminant-NEC (f-NEC), non-fulminant-NEC (nf-NEC) and No-NEC according to severity of clinical and histologic features. Of 38 piglets, 37% (n=14) developed nf-NEC, 18% (n=7) developed f-NEC and 45% (n=17) had No-NEC. There were significant differences in baseline heart rate (p=0.008), SpO_2_ (p<0.001) and A-NIRS (p<0.001) among the three groups. A-NIRS values of NEC piglets remained lower throughout the study with mean for f-NEC of 69±3.8%, 71.9±4.04% for nf-NEC, and 78.4±1.8% for No-NEC piglets (p<0.001). A-NIRS <75% predicted NEC with 97% sensitivity and 97% specificity. NEC piglets demonstrated greater variability from baseline in A-NIRS than healthy piglets (10.1% vs. 6.3%; p=0.04). Mean pI-FABP levels were higher in animals that developed NEC compared to No-NEC piglets (0.66 vs. 0.09 ng/mL;p<0.001). In f-NEC piglets, pI-FABP increased precipitously after feeds (0.04 to 1.87 ng/mL;p<0.001). *p*I-FABP levels increased in parallel with disease progression and a value >0.25ng/mL identified animals with NEC (68% sensitivity and 90% specificity). NIRS is a real-time, non-invasive tool that can serve as a diagnostic modality for NEC. In premature piglets, low A-NIRS in the early neonatal period and increased variability during initial feeds are highly predictive of NEC, which is then confirmed by rising plasma I-FABP levels. These modalities may help identify neonates with NEC prior to clinical manifestations of disease.

## Introduction

Necrotizing enterocolitis (NEC) is a serious and potentially devastating disease affecting newborn infants. The most common gastrointestinal emergency in neonates, NEC occurs in 1 infant per 1000 live births [1] and in approximately 10% of neonates with very low birth weight (between 500 and 1500g) [2]. It is a disease of significant morbidity and mortality, with mortality rates ranging from 15% to 50% and inversely proportionate to birth weight and gestational age [1, 3, 4].

Despite significant research, the exact pathophysiology of NEC remains elusive. However, it is widely believed to be multi-factorial, involving prematurity, enteral feeding, gut ischemia, and bacterial colonization. [5]. Moreover, an uncontrolled inflammatory response to bacterial colonization in the premature intestine is thought to be a unifying theory encompassing many of these factors. Due to its insidious onset, early signs of the disease are often vague and non-specific, making early diagnosis challenging [6]. Once clinical signs of NEC are apparent, in some cases the disease has advanced to a more fulminant course, making effective treatment difficult and outcomes poor.

While several risk factors for NEC have been identified, like prematurity, low birth weight, and enteral formula feeding [2, 4, 7, 8], no effective screening tool exists to accurately predict the onset of NEC within these high-risk neonates. Thus, in recent years increasing amount of the NEC research has focused on early detection, with noninvasive techniques and biological markers being some of the most promising. One such device is near-infrared spectroscopy (NIRS), which is a noninvasive method of measuring local tissue oxygen content of hemoglobin (StO_2_). Initially used in adult patients to evaluate shock and the effectiveness of resuscitation [9, 10], NIRS has also been demonstrated to effectively identify splanchnic ischemia in premature infants with NEC or other causes of acute abdomen [11]. Our group previously utilized a piglet animal model for NEC and demonstrated abdominal NIRS (A-NIRS) was not only found to show a correlation between StO_2_ and overall decreased blood oxygen content, but when applied briefly at 12-hour intervals, A-NIRS was also able to detect significantly lower StO_2_ measurements in those piglets who developed NEC vs. healthy controls, even before enteral feeds began [12].

Another promising focus of NEC research has been on the role of intestinal fatty acid binding protein (I-FABP) in the diagnosis of NEC. One of 9 types of fatty acid binding proteins, I-FABP is only expressed on mature enterocytes of the small intestine, and elevated levels of urine and serum I-FABP have been shown to accurately detect intestinal ischemia and ileal inflammation of a variety of etiologies [13, 14]. I-FABP has also been reported to be useful in diagnosing and determining severity of NEC in neonates [15, 16].

In this study we aimed to improve the effectiveness of non-invasive measures as well as biochemical markers to identify NEC early in the disease process prior to the onset of symptoms. Currently, our understanding of the temporal relationship between NEC onset and detection on diagnostic testing remains limited. We hypothesized that continuous rather than intermittent NIRS measurements of splanchnic tissue oxygenation (StO_2_) and plasma I-FABP (pI-FABP) levels used in combination would identify critical time points in the onset and progression of intestinal injury in premature piglets with necrotizing enterocolitis.

## Methods

### Ethics Statement

All procedures were approved by the Animal Care and Use Committee of and the Internal Review Board (AN-4121) of Baylor College of Medicine and conducted in accordance with the Guide for the Care and Use of Laboratory Animals [Department of Health and Human Services publication no. 85–23, revised 1985, Office of Science and Health Reports, NIH, Bethesda, MD]. All surgeries were performed under general anesthesia, and all efforts were made to minimize suffering.

### Necrotizing Enterocolitis Piglet Model

**Fig 1:**
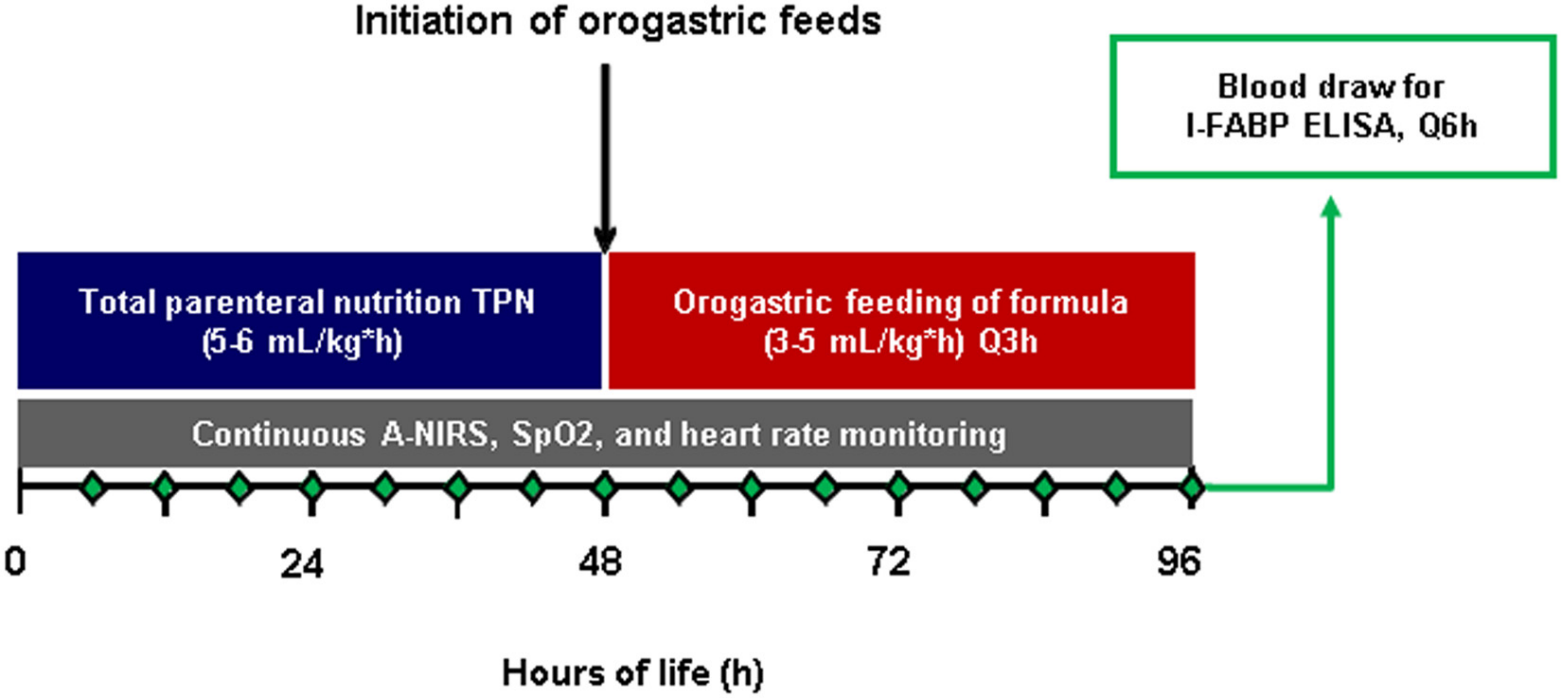
Premature Pig Model of NEC—Study Design. This is a graphic representation of our premature piglet model of NEC demonstrating timing of parenteral and enteral nutrition.

These studies were performed using a premature piglet model of spontaneous necrotizing enterocolitis (Fig 1). Premature piglets were delivered at 103 days gestation (115 days term) of four pregnant crossbred sows obtained from the Texas Department of Criminal Justice (Huntsville, TX). Sows were housed in the Children’s Nutrition Research Center and were given food and water ad libitum. Before cesarean section, sows were first injected intramuscularly with glycopyrrolate (0.01mg/kg; Baxter Healthcare Corp., Deerfield, IL) followed by a mixture of ketamine (20 mg/kg) + xylazine (2 mg/kg; Butler Schein, Dublin, OH). Then, 40–50 mL of 2% Lidocaine (Sparhawk Laboratories, Inc., Lenexa, KS) was injected along the lumbar vertebrae to induce paravertebral blockade. After endotracheal intubation, anesthesia was maintained with isoflurane inhalation (1–3% in oxygen). Under sterile conditions, a midline laparotomy incision was made and the uterus exposed. Following a hysterotomy incision on the anti-mesenteric uterine surface, preterm pigs were sequentially delivered after ligating and transecting the umbilical cord. Following delivery, piglets were immediately resuscitated by suctioning the nasopharynx free of amniotic fluid, received intermittent bag-mask ventilation to aid with respirations and were placed in temperature regulated incubators maintained at 31–32°C. Shortly after birth, all piglets were implanted with jugular venous catheters and orogastric tubes while anesthetized with isoflurane via gas mask at 5% for induction and 1–3% for maintenance. The piglets received buprenorphine (0.01 mg/kg every 12 hours intramuscularly) postoperatively for 48 h to minimize pain with the first injection given during the end of the surgical procedure prior to recovery from anesthesia and then every 12 hours thereafter. Piglets then received total parenteral nutrition (TPN) for 48 h continuously at a rate of rate of 5–6 ml/kg*h. The TPN solution contained a complete elemental nutrient mixture with amino acid, glucose, lipids and electrolytes as described previously [17, 18]. After 48 h TPN was discontinued and orogastric tube-feeding with an infant formula (3–5 ml/kg*h) every 3 h was initiated and continued for the next 48 h. The composition of the infant formula used has been reported previously [19] and yields a 40–60% incidence of NEC, thus allowing for experimental animals as well as littermate controls. Piglets that succumbed to NEC prior to the completion of the 96-h-protocol immediately underwent necropsy. Piglets that developed clinical signs of NEC including decreased activity level, feeding intolerance, abdominal distention, vomiting, diarrhea, and bloody stool and that did not improve within 6 hours of onset of symptoms were humanely euthanized. There was at least one research associate in the room at all times during the 96-hour study. Each piglet had hemodynamic parameters continuously monitored as detailed below. In addition, physical examination and assessment was performed every 3 hours prior to each feed. The piglets were housed in stainless steel cages (Dimensions: 24 inches x 12 inches x 24 inches (length x width x height)). The cages were kept in temperature and light controlled rooms. The piglets were housed at room temperatures 29–32°C. At the conclusion of the 96-h study period, the remaining piglets were euthanized and underwent necropsy and histologic tissue preparation. All piglets were euthanized with an infusion of Beuthanasia (0.8mg/kg). The clinical and pathological features of NEC in these piglets are similar to the phenotype seen in neonates.

### Near Infrared Spectroscopy and Pulse Oximetry Measurements

Neonatal NIRS probes and monitors were obtained from CAS Medical Systems Inc., (Branford, CT). A NIRS probe was placed on the piglet’s right lower quadrant of the abdomen within 2 h of birth. Next, a pulse-oximeter probe (Nellcor N-595 with MAX-N sensor; Covidien Inc., Mansfield, MA) was placed on the tail of the piglet and secured with elastic tape. The data were continuously captured every 2 seconds onto a bedside notebook computer into which all other hemodynamic parameters (heart rate and systemic arterial oxygen saturation) and clinical symptoms (activity level, feeding intolerance, abdominal distention, vomiting, diarrhea, and bloody stool) from each piglet were entered in real-time. In addition to monitoring the hemodynamic parameters, the piglets were monitored with research staff present at all times for the entire duration of the study. Post-hoc, the A-NIRS values were averaged into 1-minute periods to ease the determination of differences in splanchnic tissue oxygen saturation between piglets stratified by NEC severity. To evaluate peri-prandial variability related to enteral feeding, A-NIRS measurements of 15-minute segments just before and after feeding were analyzed. The average percent change from baseline was calculated to determine StO_2_ variability following the first two enteral feeds.

### Intestinal Fatty Acid Binding Protein (I-FABP) and Serum Amyloid A (SAA) Measurements

Umbilical cord blood was obtained from each piglet at birth and immediately after placement of internal jugular venous catheters to establish baseline levels of I-FABP and to determine any birth-related effects on intestinal well-being. Piglets then had blood drawn from the indwelling internal jugular venous catheters at 6-h intervals for the duration of the study. Heparinized blood samples were centrifuged to isolate the plasma used for SAA and I-FABP assays, both of which have been studied as biomarkers of NEC. For SAA analyses we utilized a commercially available multispecies SAA sandwich ELISA kit (Tridelta Development Ltd., Maynooth, Co. Kildare, Ireland). The I-FABP assays were performed using a commercially available anti-human I-FABP sandwich ELISA (Hycult Biotech Inc, Plymouth Meeting, PA), which has proven very sensitive in measuring changes in porcine I-FABP in our prior experiments. After necropsy, western blot analyses were also conducted on proximal jejunum samples to measure the amount of I-FABP content remaining in the tissue samples at the time of death. Select samples of NEC and No-NEC proximal jejunum tissue also underwent immunohistochemical fluorescent staining using NorthernLights 557 Fluorochrome-labeled Donkey Anti-Rabbit IgG antibody (R&D Systems, Minneapolis, MN).

### Necropsy and Histologic NEC scoring

**Fig 2:**
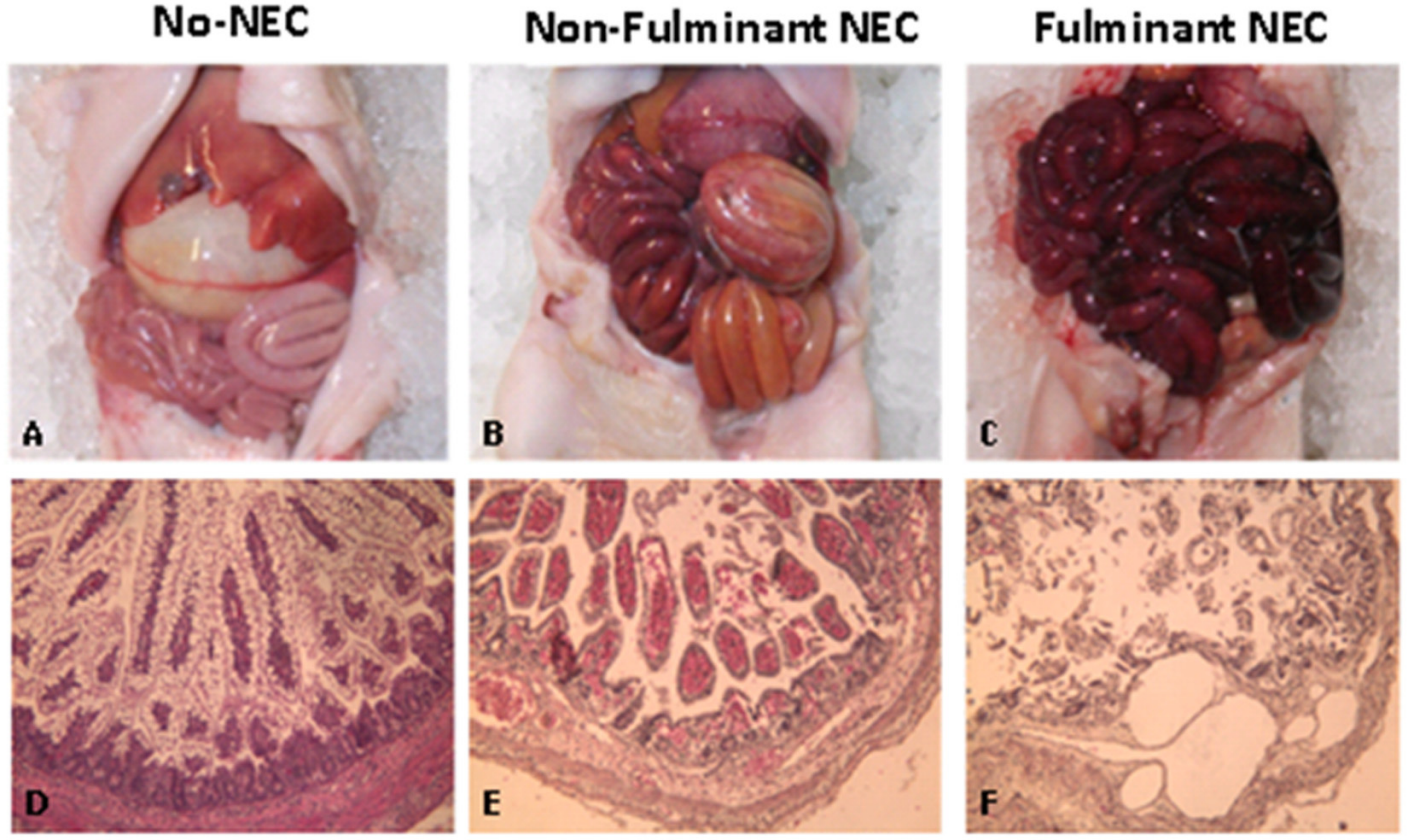
Premature Pig Model of NEC—Gross and Histological Examination. A, Gross examination of the abdominal viscera of healthy No-NEC piglet. B, Gross examination of piglet with non-fulminant NEC shows inflamed and congested small bowel with areas of focal necrosis. C, Gross examination of piglet with fulminant NEC shows diffuse necrosis throughout the entire bowel. D, Histologic examination of the normal small intestine of a No-NEC piglet. E, Histologic examination of the small intestine of a piglet with non-fulminant NEC demonstrating moderate mucosal injury, blunting of villi and separation of the basement membrane. F, Histologic examination of the small intestine of a piglet with fulminant NEC demonstrating severe mucosal injury and diffuse pneumatosis.

Necropsy was performed on all animals immediately after death for NEC piglets and after euthanasia for piglets completing the 96-h study period. Tissue samples were harvested from the stomach, the liver and the small bowel at proximal, middle, and distal regions between the proximal jejunum and colon for histologic analysis. Classification of NEC severity was based on a combination of clinical, histologic and pathological features as previously described [12]. Piglets were classified as fulminant-NEC (f-NEC), non-fulminant-NEC (nf-NEC) and No-NEC according to the severity of clinical and histologic features (Fig 2).

### NIRS Validation Study

The CAS Medical Inc. NIRS system is FDA cleared for use in neonates. However, to validate the system in premature piglets, five piglets were utilized to validate the NIRS values against actual systemic and splanchnic oxygenation. Piglets had an umbilical arterial catheter (UAC), an umbilical vein catheter (UVC) and an orogastric feeding tube placed. The UAC tip placement was below the renal arteries and the pressure was transduced in real time. The UVC tip placement was just distal to the ductus venosus to sample the confluence point of mesenteric venous blood. Piglets were intubated with an uncuffed neonatal endotracheal tube and mechanically ventilated with a volume ventilator (Ohmeda 7810, Madiscon, WI) to the point of controlled respiration with a constant PEEP setting of 2 (~ 6-7ml/kg at 25 breaths per minute). The controlled minute ventilation was maintained to minimize fluctuations in PaCO_2_ and resultant dynamics in cerebral perfusion. The piglets then had NIRS probes placed, one on the head and another on the right lower quadrant, as well as a pulse-oximeter probe on the tail. Abdominal and cerebral NIRS readings were then continuously recorded on a netbook computer. Piglets were given 4 incremental steps of FiO_2_ with a mix of oxygen and balance of nitrogen: first ambient air at 0.21 followed by 0.60, 0.08 (sub-ambient), and 1.0 FiO_2_. During each level, 0.3ml of whole blood was drawn into heparinized 1ml syringes from the UAC and UVC simultaneously to obtain SaO_2_ and ScvO_2_ measurements and immediately underwent CO-oximetry analysis with a hemoximeter (Model OSM-3; Radiometer Medical A/S, Copenhagen, Denmark) for functional oxygenation (S_ref_O_2_).

Since meconium is a chromophore that can interfere with NIRS absorptive capacity in the abdomen, piglets then had their gastrointestinal tract flushed with a normal saline enema to induce meconium passage. This allowed for assessment of abdominal NIRS data to derive a calibration value for different absorptive patterns before and after meconium clearance. The four plateaus of oxygenation and CO-oximetry data in each piglet as well as A-NIRS data with and without meconium were analyzed against reference calculations from blood samples using a Pearson correlation. The resultant algorithm was then utilized to characterize NIRS performance for a calibration factor to help analyze the remainder of the piglet NIRS readings.

### Statistical Analysis

All statistical analyses were performed using SPSS v. 21 (IBM Corporation, Armonk, New York). The Shapiro-Wilk test was applied to test for normality. Data with normal distribution were analyzed using analysis of variance (ANOVA) for repeated measures with post-hoc analysis. Data with non-normal distribution were analyzed using Kruskall-Wallis non-parametric testing. We also performed receiver operating characteristic (ROC) curves to determine appropriate cut-off values for StO_2_ and pI-FABP levels. Results are expressed as means and standard errors of the mean (SEM) unless otherwise specified. A p-value of <0.05 was considered statistically significant.

## Results

### Necrotizing Enterocolitis and Baseline Vitals

Forty-six premature piglets were delivered from 4 litters. Two piglets died prematurely at approximately 60 h of life due to non-NEC etiology and were excluded from analysis. One piglet was subjected to the same experimental conditions but was not monitored throughout the study due to limited NIRS equipment. Five piglets were utilized for NIRS validation studies and consequently were not subjected to the experimental conditions. The remaining 38 piglets were included in the study cohort and had complete data available for analysis, 19 (50%) of which survived the full 48 hours of enteral feeding. All animals were exposed to the same experimental conditions and were sub-categorized post-hoc into groups based on NEC severity.

Seventeen piglets (45%) had no clinical signs of NEC and on histologic analysis had uniformly pink and viable small and large intestines with no evidence of inflammation, necrosis or pneumatosis. These piglets were assigned as the No-NEC group. Twenty-one (55%) piglets developed NEC, of those 7 developed fulminant NEC, that all demonstrated clinical symptoms of NEC within 6 h of initiation of enteral feeds and all succumbed to their disease by 18 h after initiation of feeds. On histologic analysis, this group had widespread necrosis and pneumatosis throughout the length of the bowel. Fourteen piglets developed non-fulminant NEC, which was characterized by a more gradual clinical course. These piglets demonstrated clinical signs of NEC 18–24 h following the initiation of enteral feeds and five survived the duration of the study until sacrifice at 96 h of life. The histologic analysis of this group demonstrated more focal evidence of inflammation and necrosis, some limited to only portions of the small bowel, others only to the large intestine (Fig 2).

When examining the groups of piglets at baseline based on NEC severity, there were no differences in birth weight (p = 0.39) or baseline hematocrit (p = 0.25) between the three groups (Table 1). However, there were significant differences in baseline heart rate (bpm, p = 0.008), systemic oxygen saturation (SpO_2_, p<0.001) and A-NIRS values (StO_2_, p<0.001) among the three groups of piglets. The f-NEC piglets on average had baseline heart rates (163 ± 14.8 bpm) similar to the nf-NEC piglets (166 ± 31.6 bpm), which were both significantly higher when compared to the Non-NEC piglets (135 ± 29.2 bpm) (Table 1).

**Table 1:** Baseline measurements of piglets by NEC severity.

	Fulminant NEC (n = 7)	Non-fulminant NEC (n = 14)	No-NEC (n = 17)	P-value
Birth weight (g)	1170 ± 266	1046 ± 192	1031 ± 242	0.39
Baseline Hematocrit	22.8 ± 2.5	20.9 ± 2.7	19.6 ± 5.6	0.25
Baseline HR (bpm)	163 ± 14.8	166 ± 31.6	135 ± 6.9	**0.008**
Baseline SpO2 (%)	53.1 ± 9.3	77.5 ± 1.8	89.4 ± 8.0	**<0.001**
Baseline A-NIRS (%)	64.9 ± 6.7	74.2 ± 6.8	79.5 ± 7.6	**<0.001**

NEC—necrotizing enterocolitis; HR—heart rate; bpm—beats per minute; SpO2—oxygen saturation; A-NIRS—abdominal near infrared spectroscopy.

### Heart Rate

**Fig 3:**
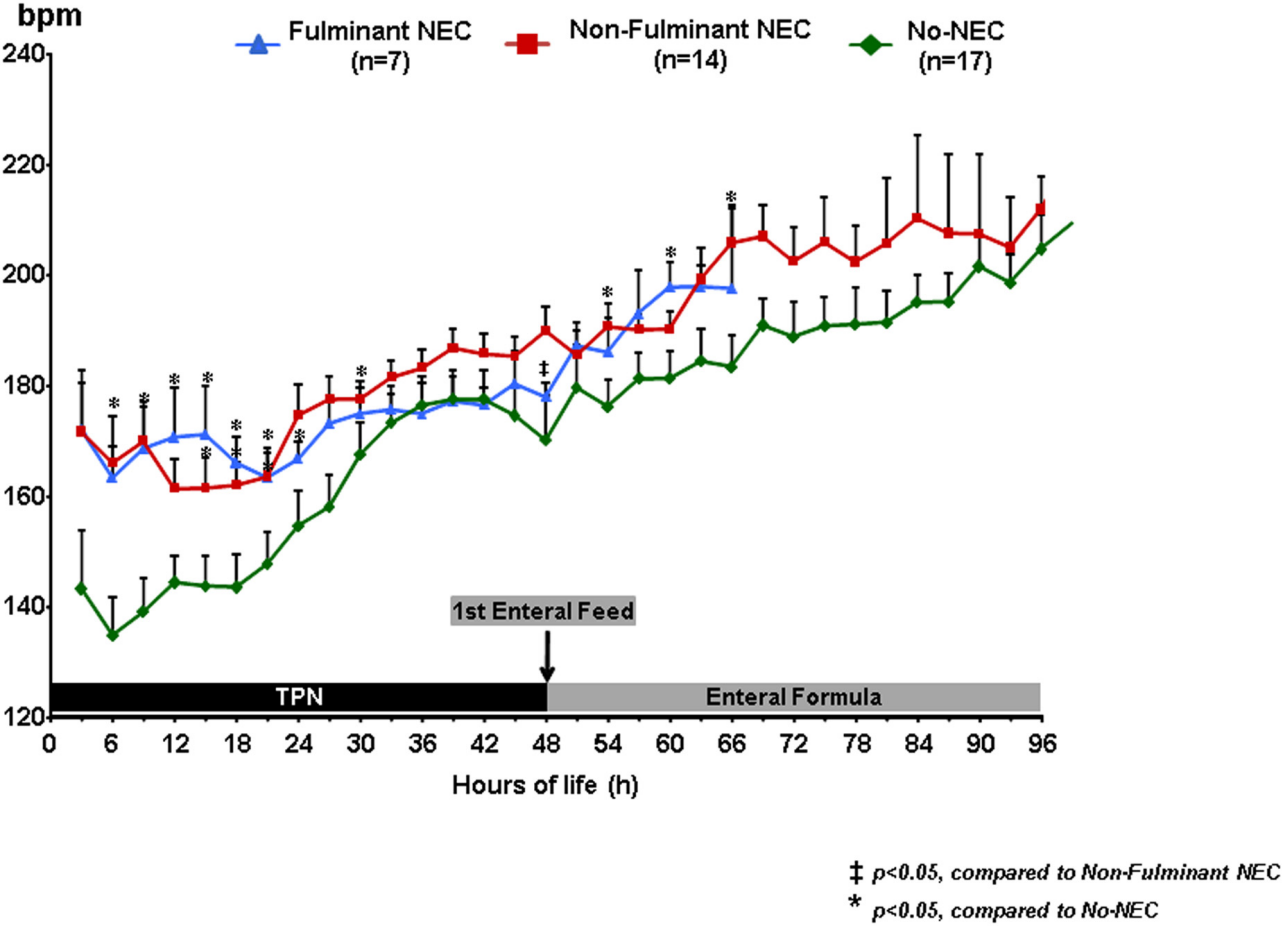
Continuous Heart Rate Measurements. Heart rate data stratified by NEC severity groups demonstrating an overall increasing trend in heart rates throughout the study period and that f-NEC and nf-NEC piglets were significantly more tachycardic during the first 24 hours of life than No-NEC piglets. *‡ = p<0*.*05*, compared to Non-Fulminant NEC; * = p<0.*05*, *compared to No-NEC*.

All three groups demonstrated an increasing trend in heart rate throughout the duration of the study; however, the NEC piglets were significantly more tachycardic during the first 24 h of life (Fig 3). The f-NEC and nf-NEC piglets had heart rates ranging from 163–175 bpm during this time period whereas the No-NEC piglets ranged from 135–158 bpm. During the following 24 h heart rates were comparable between the three groups. After the initiation of enteral feeds at 48 h of life, the NEC piglets had a greater increase in mean heart rate (20% increase, 185 to 222 bpm) than the No-NEC group (16% increase, 180 to 209 bpm) likely in response to an increased metabolic demand. The average heart rate throughout all time points for f-NEC piglets was 178 ± 11 bpm, 189 ± 17 bpm for nf-NEC piglets, and 175 ± 21 bpm for No-NEC piglets (p = 0.003). On post-hoc analyses, the differences were only significant between the nf-NEC piglets and the other two groups. The difference between the f-NEC and No-NEC piglets was not statistically significant.

### Systemic Oxygen Saturation (SpO_2_)

**Fig 4:**
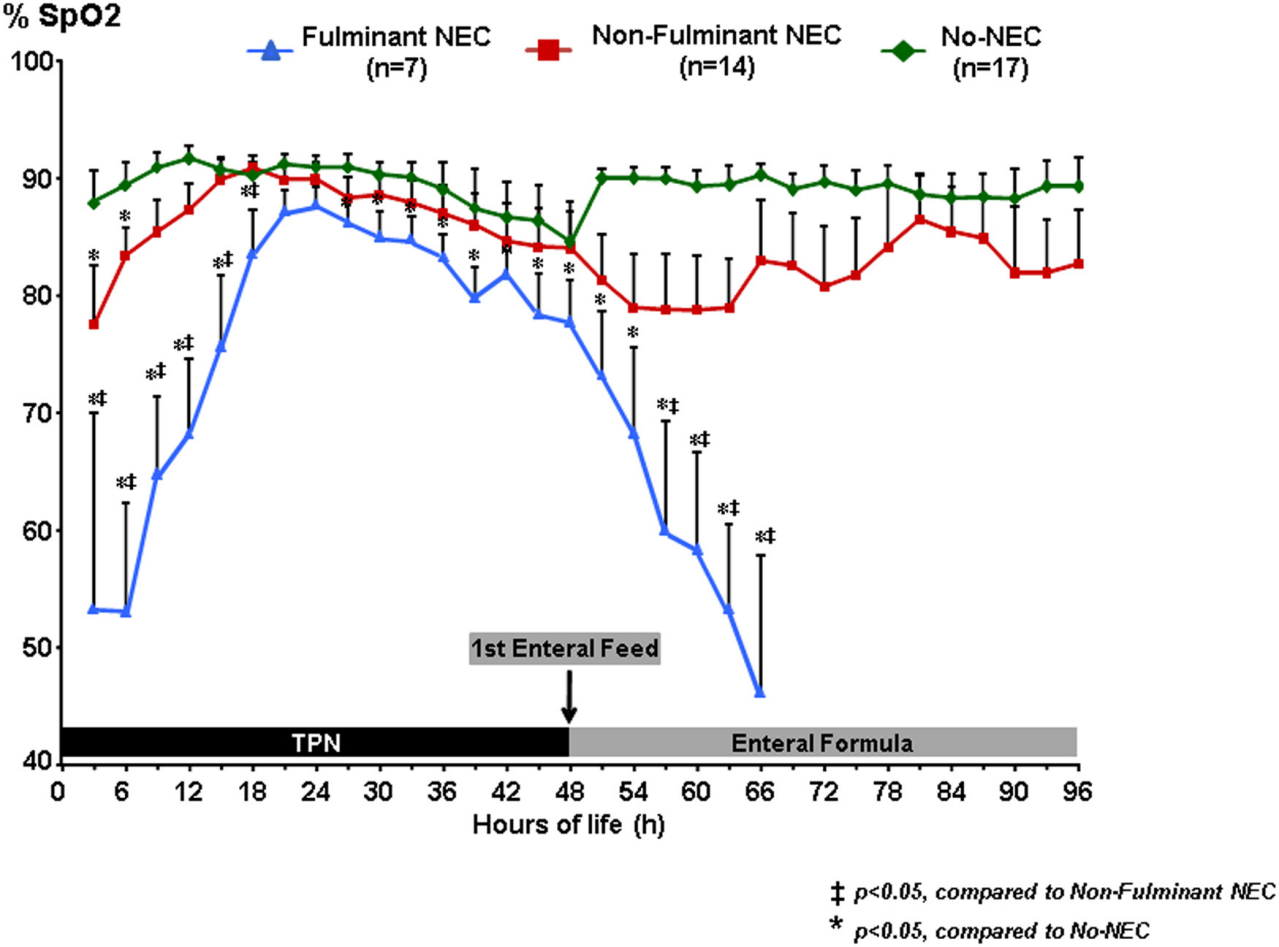
Continuous Systemic Oxygen Saturation (SpO_2_) Measurements. Systemic oxygen saturation data stratified by NEC severity groups demonstrating that f-NEC piglets were significantly more hypoxic at baseline and for the first 18 hours of life until the normalized at 21 hours of life, and nf-NEC were only more hypoxic than No-NEC piglets during the first 3 hours of life. *‡ = p<0*.*05*, compared to Non-Fulminant NEC; * = p<0.*05*, *compared to No-NEC*.

Systemic oxygen saturation was significantly different across all three piglet groups at baseline (p<0.001). The fulminant-NEC group had the lowest levels of oxygen saturation during the first 15 h of life, which was significantly lower than both the No-NEC group (p<0.001) and the piglets who went on to develop nf-NEC (p = 0.015) (Fig 4). There was a steep upward curve during this time period likely reflecting improvements in oxygen saturation following resuscitation and increased arousal as the effects of sedation diminished. By 18 h of life, the SpO_2_ was comparable for all three groups, ranging from 87% in the f-NEC group to 91% in the No-NEC group. After the 24 h of life, the f-NEC group began to demonstrate a downward trend in systemic oxygenation, and this downward slope increased dramatically after the initiation of feeds at 48 h, and levels became significantly different from both nf-NEC and No-NEC groups by 54 h of life. In contrast, the nf-NEC piglets only had significantly lower SpO_2_ than the No-NEC piglets during the first 3 h of life, and by 15 h of life the SpO_2_ levels were equivalent. The SpO_2_ levels for nf-NEC and No-NEC piglets paralleled each other for the remainder of the study, although there was a slight divergence after the initiation of enteral feeds. The average SpO_2_ throughout all time points for f-NEC piglets was 72% ± 13%, 84% ± 3.7% for nf-NEC piglets, and 89% ± 1.5% for No-NEC piglets (p<0.001). On post-hoc analyses, the overall mean differences remained significant between all three groups when compared to one another.

### Abdominal NIRS (StO_2_)

**Fig 5:**
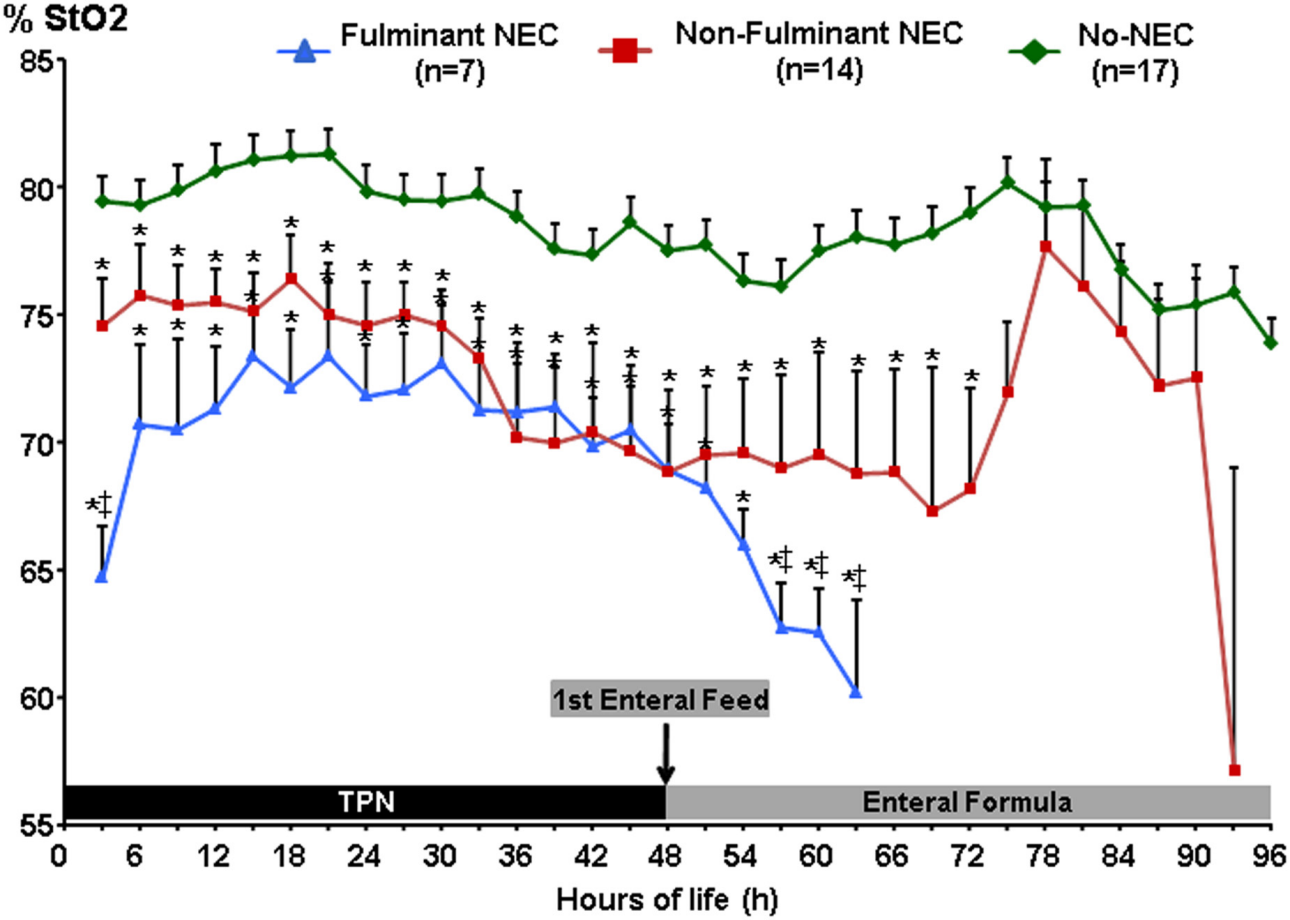
Continuous Abdominal NIRS-Tissue Oxygen Content of Hemoglobin (StO_2_) Measurements. Abdominal NIRS data stratified by NEC severity groups demonstrated that f-NEC piglets had significantly lower A-NIRS values than both the nf-NEC and No-NEC groups at baseline, and then both NEC groups maintained significantly lower A-NIRS values than No-NEC piglets throughout the majority of the study. *‡ = p<0*.*05*, compared to Non-Fulminant NEC; * = p<0.*05*, *compared to No-NEC*.

A-NIRS values were also significantly lower in animals that developed NEC compared to the No-NEC group, and remained lower throughout the study period (p<0.001) (Fig 5). The f-NEC group had the lowest A-NIRS baseline values. Despite the systemic oxygen saturation normalizing to levels comparable to the No-NEC piglets, the same was not true for A-NIRS, which never reached above 73%. A similar pattern was seen in the nf-NEC group, where A-NIRS values were also significantly lower than No-NEC piglets and levels never reached above 76% during the first 48 h of life, despite the SpO_2_ normalizing. At 36 h of life A-NIRS values diverged even greater from the healthy piglets and reached a trough of 67% approximately 24 h after the initiation of feeds. The average A-NIRS throughout all time points for f-NEC piglets was 69% ± 3.8%, 71.9% ± 4.04% for nf-NEC piglets, and 78.4% ± 1.8% for No-NEC piglets (p<0.001). On post-hoc analyses, the overall mean differences remained significant between the f-NEC and No-NEC piglets (p<0.001) and between nf-NEC piglets and the No-NEC group (p<0.001), but there was no difference between the two subgroups of NEC piglets (p = 0.087). On ROC curve analysis an A-NIRS < 75% identified animals progressing to NEC with 97% sensitivity and 97% specificity.

To assess the variability of A-NIRS readings in response to enteral feeding, peri-prandial StO_2_ values were analyzed to calculate the percent change from baseline for each piglet following the first two enteral feeds. NEC piglets demonstrated significantly greater variability from baseline in A-NIRS values than healthy piglets (10.1% vs. 6.3%, p = 0.04). This variability in A-NIRS was detected prior to the onset of clinical signs of NEC, which may suggest a predisposition to poor regulation of the splanchnic vasculature in piglets that developed NEC.

### NIRS Validation Study

The NIRS-derived SctO_2_ values were calibrated against SaO_2_ and ScvO_2_ blood samples from five piglets that underwent validation testing. The progressive sequence consisted of 4 progressive steps with inspired oxygen concentrations at 21%, 60%, 8% and 100% FiO_2_. The ratio of the volume of arterial blood (SaO_2_–30%) to venous blood (ScvO_2_–70%) used to calculate A-NIRS yields the following reference algorithm from the following equation where REF CX represents the values obtained from the CO-oximetry analysis:

$$Corrected\;StO_{2}vs.\;REF\;CX\;(0.3*SaO_{2}\;+\;0.7*ScvO_{2})$$

**Fig 6:**
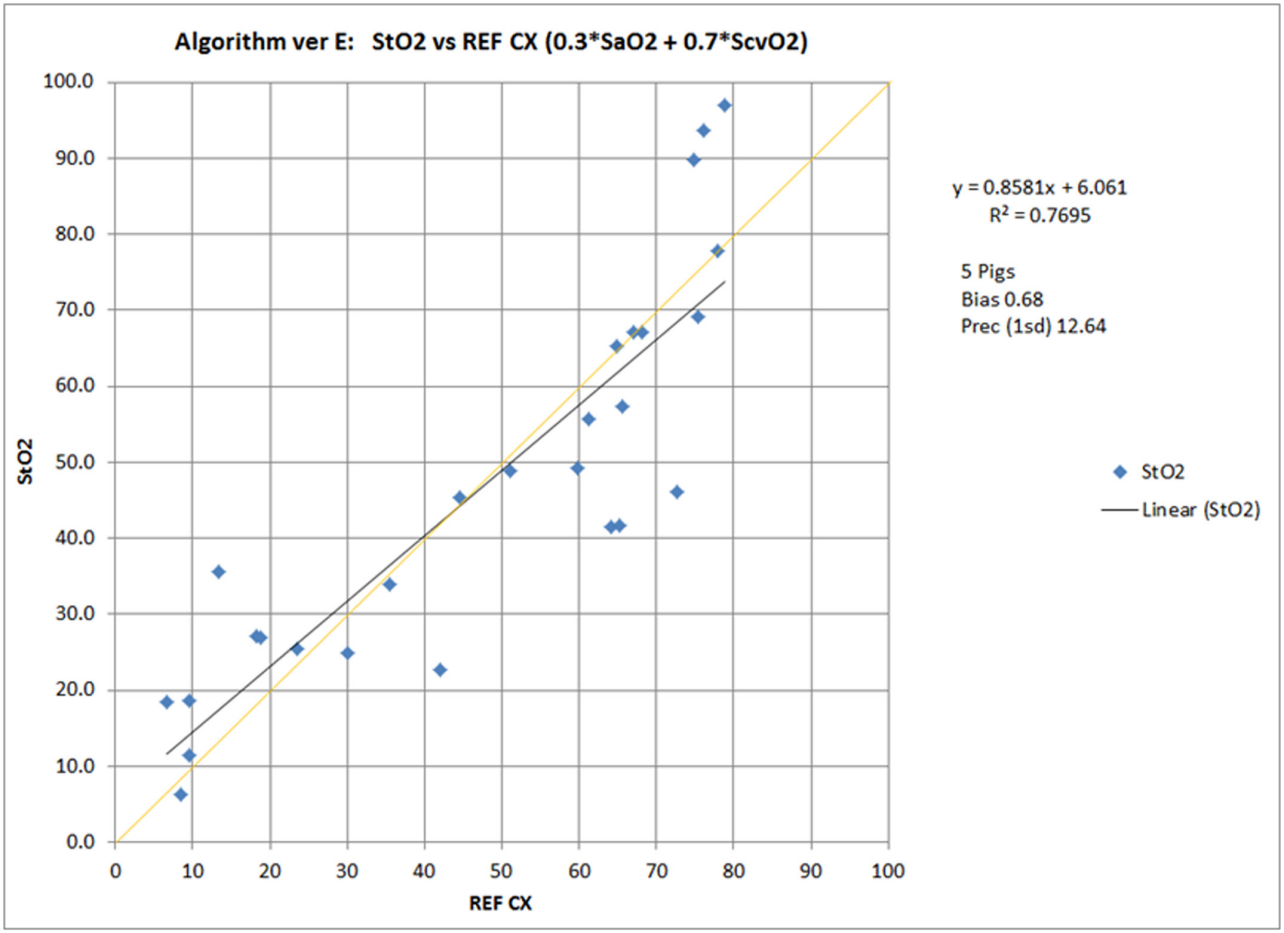
NIRS Validation Study. A Pearson correlation examining StO2 values and reference calculations from blood draws demonstrated a strong correlation between measured NIRS and actual oxygen saturation levels (R^2^ = 0.7695).

A Pearson correlation examining StO_2_ values and reference calculations from blood draws demonstrated a strong correlation between measured NIRS and actual oxygen saturation levels (R^2^ = 0.7695) (Fig 6).

### Intestinal Fatty Acid Binding Protein and Serum Amyloid A

**Fig 7:**
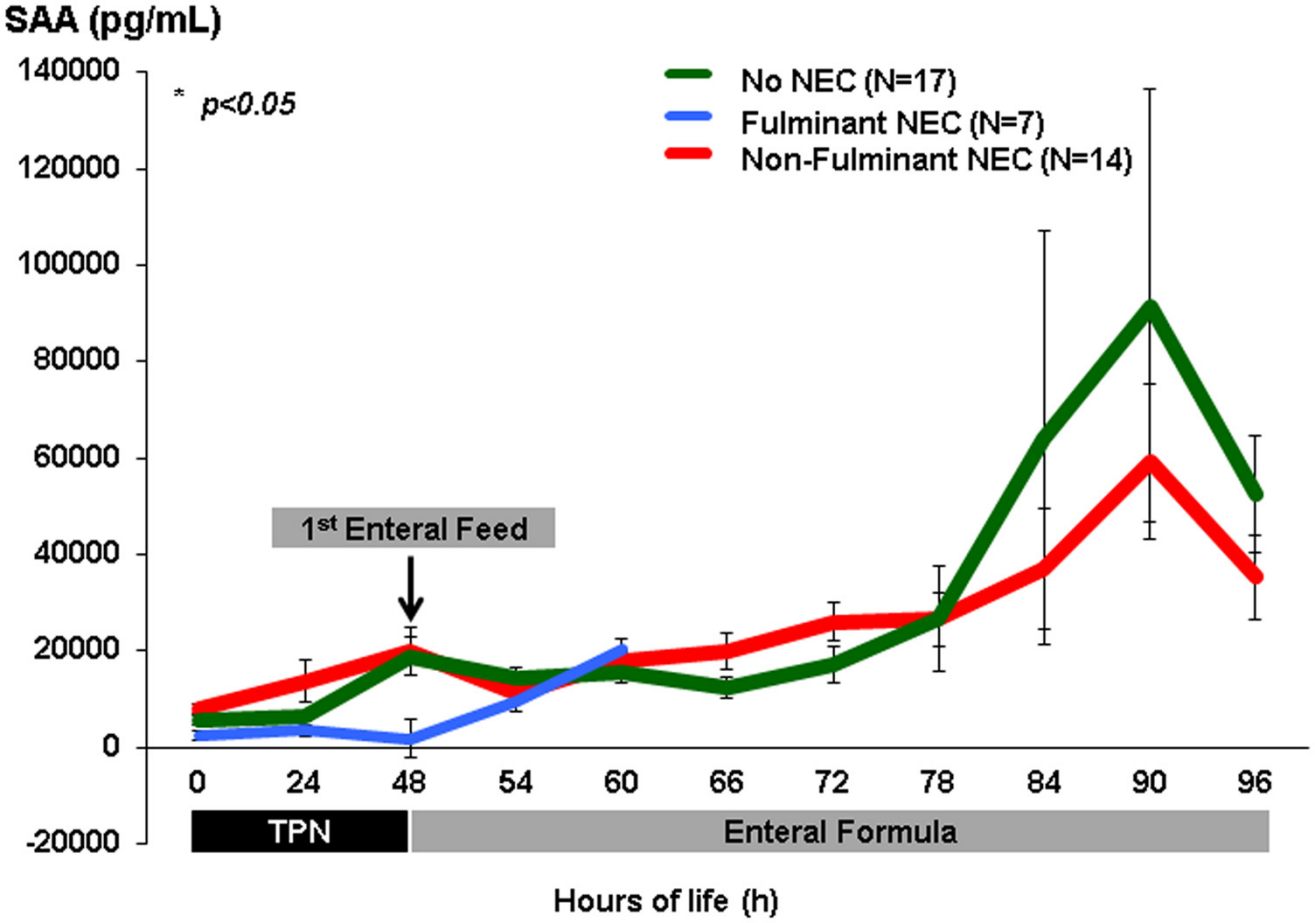
Serum Amyloid A (SAA) in Plasma. Plasma SAA values stratified by NEC severity groups demonstrating a gradual rise in all piglets, particularly after the initiation of enteral feeds, but no significant differences between the three experimental groups.

**Fig 8:**
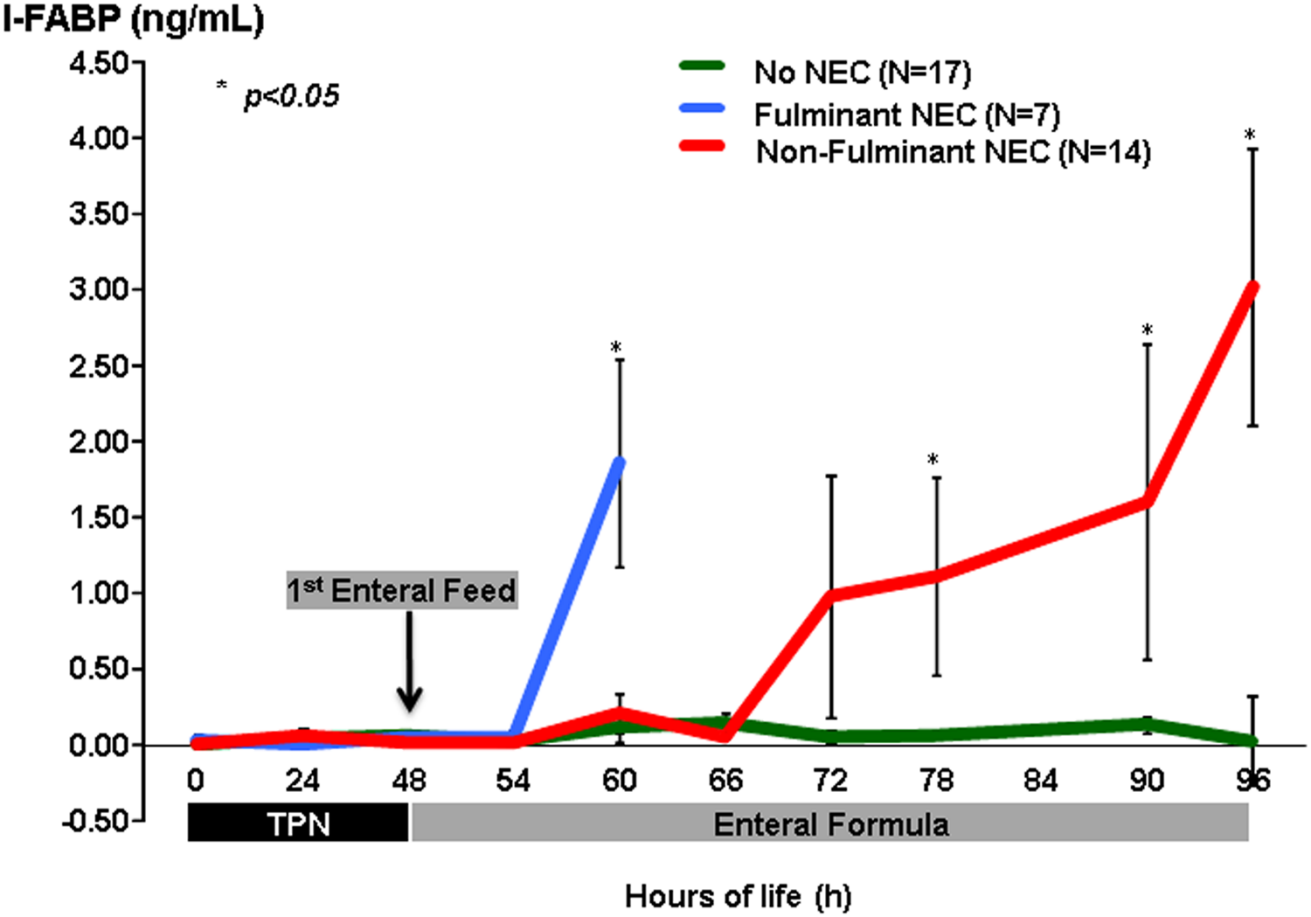
Intestinal Fatty Acid Binding Protein (I-FABP) in Plasma. Plasma I-FABP values stratified by NEC severity groups demonstrating a precipitous rise in the f-NEC group shortly after the initiation of feeds and a more gradual rise in the nf-NEC group that paralleled clinical disease progression. The No-NEC piglets never had an appreciable rise in their serum I-FABP.

During the first 48 h of the study while piglets were strictly on TPN and prior to enteral feeds, there were no appreciable levels of plasma I-FABP and low levels of SAA detected, indicating minimal intestinal tissue injury. Once enteral feeds were initiated SAA levels increased progressively in all piglets following feeds but there was no significant difference between the three groups (Fig 7), while pI-FABP levels were significantly higher in animals that developed NEC compared to healthy piglets (0.66 vs. 0.09 ng/mL, p<0.001). When stratified by NEC severity, in piglets that developed fulminant disease, pI-FABP increased precipitously after feeds (0.04 to 1.87 ng/mL; p<0.001) and the spike was observed following the 3rd feed (Fig 8). The non-fulminant NEC group had a slower disease progression, with most exhibiting the first signs of NEC about 18–24 h after the initiation of feeds. This phenotype of the disease was also reflected in a more gradual increase in pI-FABP levels (0.01 to 3.03 ng/mL; p<0.001), and levels increased in parallel with disease progression. Although levels reached a higher peak than the fulminant NEC group, we suspect given the steep slope of the pI-FABP in the f-NEC group, peak levels would have been much higher but those piglets succumbed to their disease earlier. The No-NEC piglets never demonstrated any significant rise in pI-FABP levels throughout the duration of the study. On ROC curve analysis we identified a cutoff value of pI-FABP > 0.25 ng/mL, which identified animals progressing to NEC with 68% sensitivity and 90% specificity.

**Fig 9:**
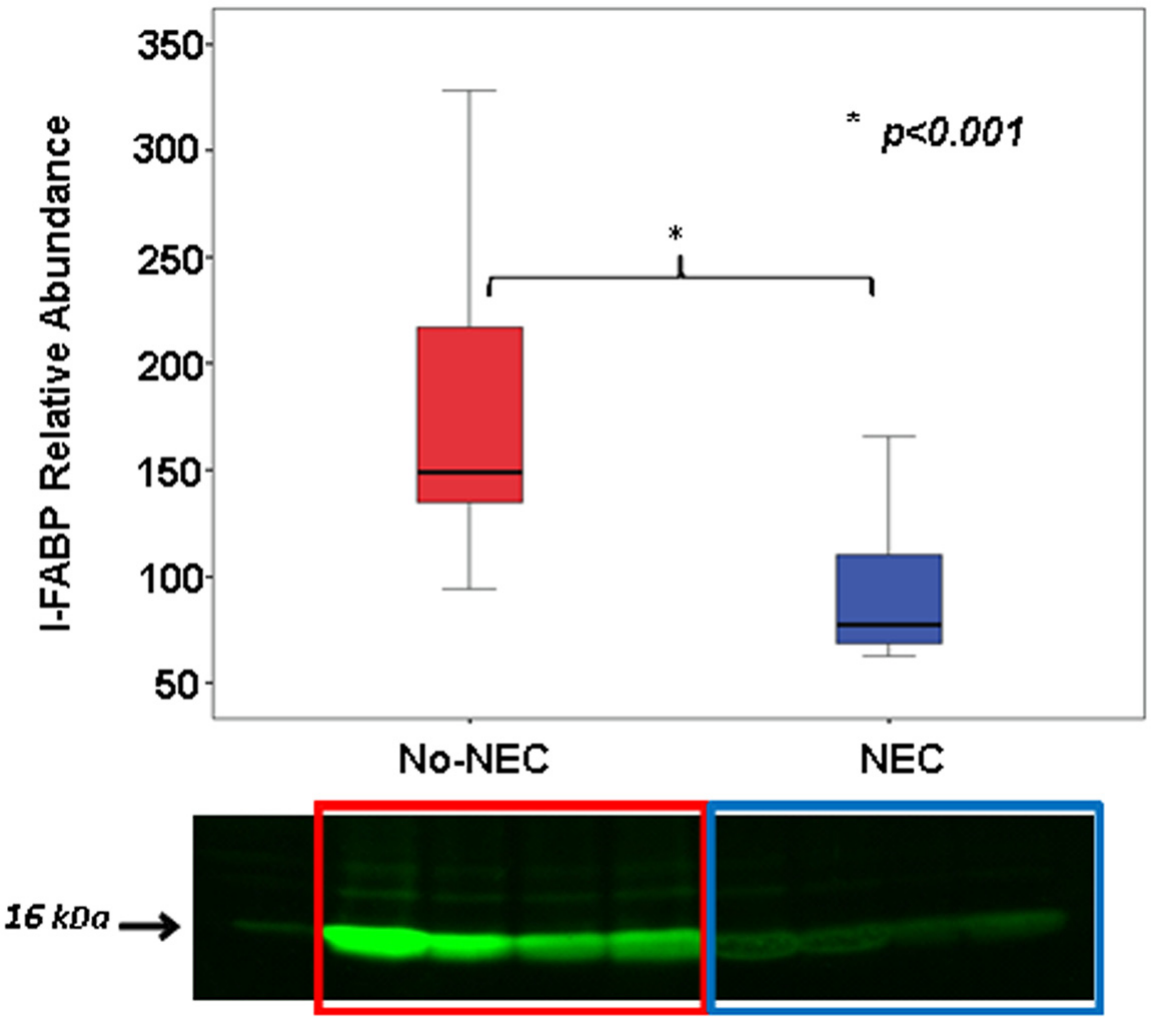
Proximal Jejunum Intestinal Fatty Acid Binding Protein (I-FABP) Abundance. Western blot data of the proximal jejunum specimens demonstrating that No-NEC piglets had significantly’ higher densitometry readings than NEC piglets.

**Fig 10:**
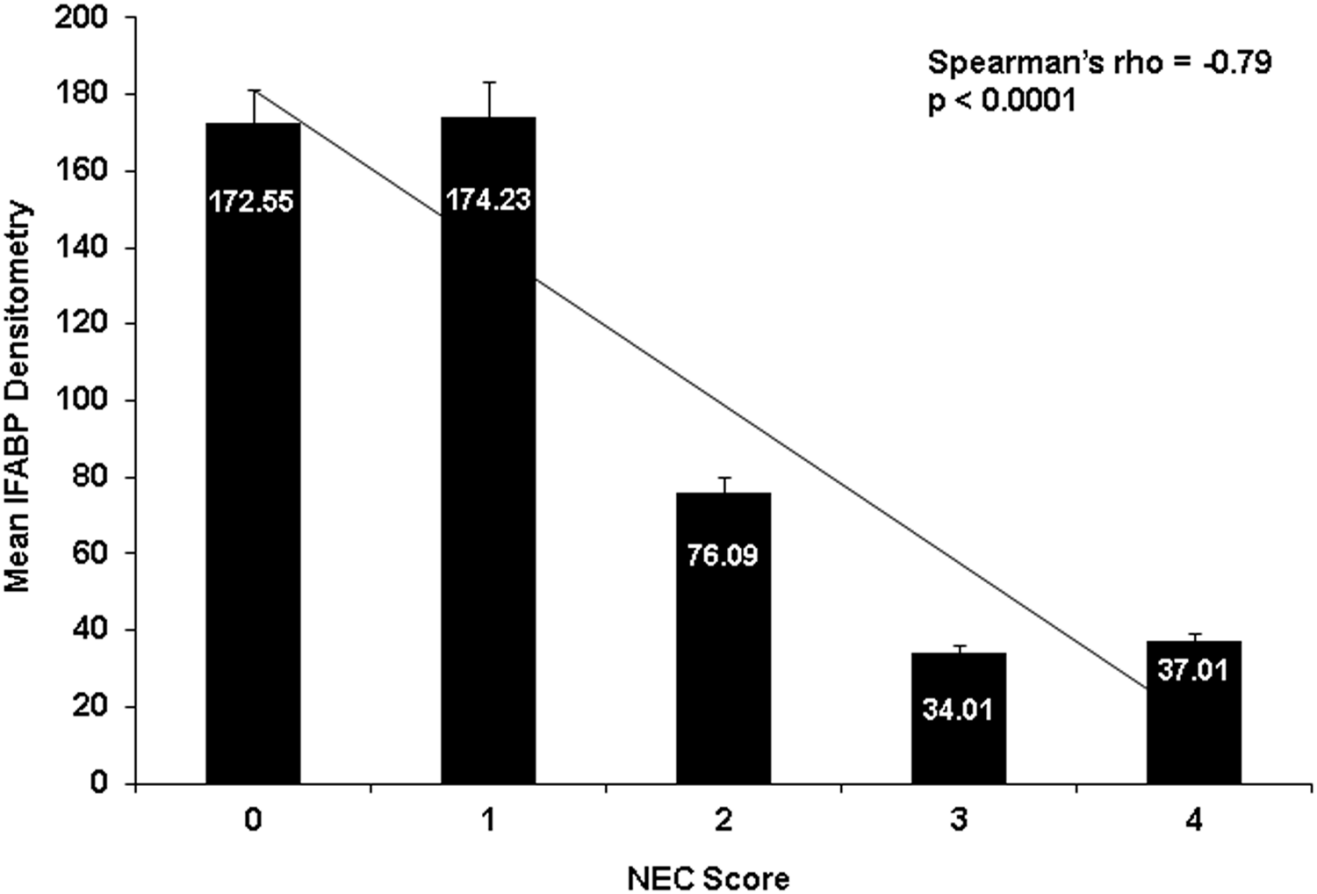
Mean I-FABP Densitometry by NEC Score. Mean I-FABP densitometry readings demonstrating an inverse correlation between the Histologic NEC score and I-FABP protein expression in proximal jejunum tissue samples by western blot.

**Fig 11:**
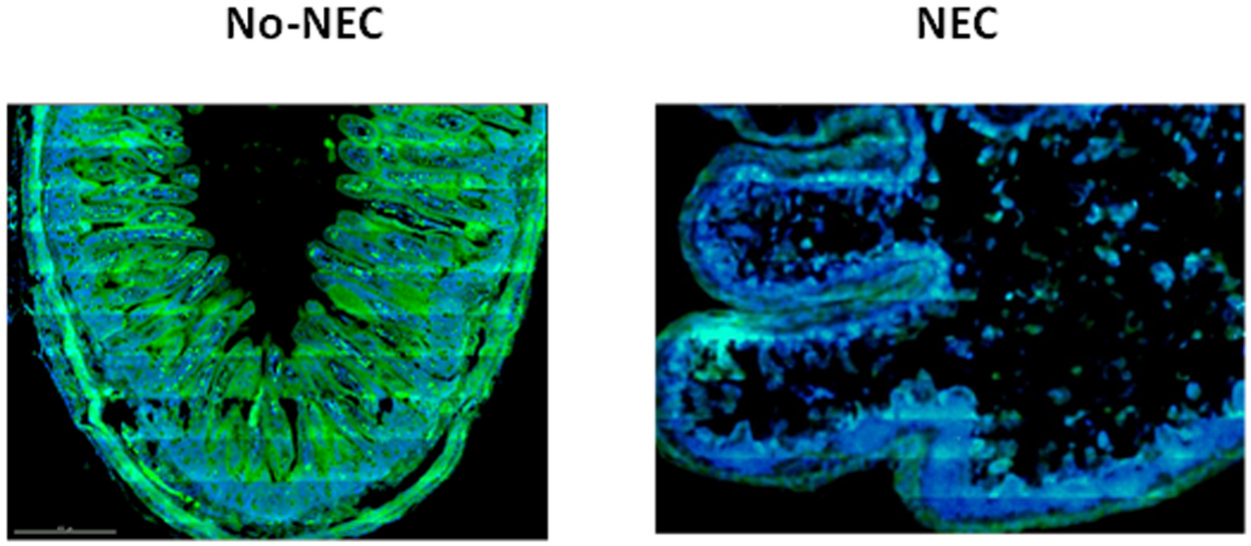
Proximal Jejunum Intestinal Fatty Acid Binding Protein (I-FABP) immunohistochemical staining. Immunohistochemistry fluorescent staining for I-FABP in proximal jejunum specimens. In these images I-FABP is represented in green. In the No-NEC piglets there is robust expression of I-FABP and in the NEC piglets the mucosa has been significantly denuded and I-FABP signal degraded.

To assess whether the elevated I-FABP detected in the plasma originated from necrotic intestinal villi we performed I-FABP western blot analyses of proximal jejunum samples, which demonstrated NEC piglets had significantly lower amounts of I-FABP protein remaining in the intestinal villi at the time of death when compared to the No-NEC group (Fig 9). To more closely examine this relationship we utilized a Spearman’s rho analysis, which revealed a strong inverse correlation between mean tissue I-FABP densitometry levels and corresponding histologic tissue injury NEC scores (rs = -0.79, p< 0.001) (Fig 10). Further corroborating the western blot results, immunofluorescent histochemical staining revealed a strong I-FABP signal in the No-NEC jejunal tissue samples and a weak signal in the NEC tissue samples (Fig 11).

## Discussion

In this study we have demonstrated that low abdominal NIRS values in the immediate perinatal period after birth are highly predictive of NEC in a premature piglet model. These findings suggest that an important element in the pathophysiology of NEC may be present and detectable at birth. We also demonstrated that plasma I-FABP levels correlate with disease severity, and levels rise with the progression of NEC in premature piglets. These modalities used together may translate to the bedside and prove useful in identifying critical time points and estimating degree of intestinal injury in neonates with NEC.

There is a compelling unmet need to establish a means for early identification of neonates at risk for NEC, even prior to the onset of symptoms, to permit trial of interventions to prevent the disease or reduce its severity. The pathogenesis of NEC involves both an ischemic and an inflammatory component, and it is likely that hypoxia that is secondary to the immaturity of the premature vascular system is the initial insult resulting in loss of gut mucosal integrity and bacterial translocation [20, 21]. As we gain a better understanding of this pathophysiology, it has been theorized that diminished blood flow and oxygenation may occur prior to the necrotic damage. These changes may be manifested in the supplying arterial network to the gut or in the portal system, which provides drainage and nutrient flow after absorption by the intestinal mucosa. Changes in perinatal blood flow have been shown to correlate with increased incidence of NEC, however it is unclear if changes in the mesenteric blood flow necessarily predispose to NEC and to what degree perfusion must change in order to increase susceptibility to NEC [22]. The temporal relationship to necrotic change in the bowel is also unclear and it is probable the physiology of both the mesenteric arterial and portal venous vessel networks are aberrant in necrotizing enterocolitis.

In this study we utilized NIRS technology to non-invasively quantify changes in intestinal oxygenation in a manner to allow for predicting necrotizing enterocolitis before it begins. Our post-hoc analysis of all pigs demonstrated piglets progressing to NEC had much lower A-NIRS values immediately after birth and remained persistently lower well in advance of the development of clinical disease. This finding provides novel evidence confirming the idea that hypoxia, caused by poor perfusion and/or increased tissue oxygen consumption, is a critical and necessary element in the pathophysiology that may begin prenatally to ultimately predispose piglets to NEC. We were also able to risk stratify the piglets by NEC severity such that piglets with the fulminant phenotype had the lowest splanchnic StO_2_ measurements, followed by the non-fulminant piglets and the No-NEC piglets had consistently higher and more stable measurements. Additionally it appears that even after systemic oxygen saturation levels normalized, A-NIRS remained highly sensitive and specific in predicting NEC piglets. This difference in systemic vs. A-NIRS values can be attributed to the measures of StO_2_ based on the composite of the weighted arterial and venous functional oxygen saturations (30% and 70%, respectively) (Fig 6) while systemic PaO_2_ levels are based solely on fluctuations in arterial blood oxygen content. In the event that mesenteric metabolic demands are low, the degradation from SaO_2_ values in abdominal NIRS is small so near systemic values can be achieved. However, PaO_2_ values are effectively decoupled from A-NIRS values in times of metabolic load (e.g., enteral nutrition uptake, low cardiac output and anemia) and can be significantly lower due to the venous component (0.70) representing the majority of the calculated NIRS value. A mechanism for monitoring the phenomena in real-time is use of the NIRS cerebrosplanchnic oxygenation ratio with the boundary value of ischemia set in human neonates at a ratio < 0.75 [11]. Thus, the expected differential depends upon the effects of physiologic activity and consequences of ischemia. In most other experimental animal models of NEC, hypoxia is an important component necessary to induce NEC, yet human NICU patients are never permitted to remain hypoxic for prolonged periods of time. Neonates are quickly resuscitated and provided supplemental oxygen as necessary. Therefore, to more closely reflect clinical practice in future studies we will treat hypoxic piglets with supplemental oxygen to verify whether this can reduce the disease pathophysiology.

The second component of our study was to incorporate a plasma biomarker as a confirmatory test for NEC. We measured serum amyloid A (SAA), a biomarker implicated in the inflammatory response that accompanies NEC. We could not discriminate between piglets that developed NEC or not based on SAA levels. We also elected to use I-FABP since elevated levels in plasma and urine have been demonstrated to indicate intestinal injury in animal models of NEC [23] and preterm infants with NEC [24, 25]. I-FABP measured in pooled urine has shown benefit in assessing protein excreted over a long period to enhance sensitivity, but is limited by decreased urine output that typically occurs with critically ill patients. Assays of pooled urine may also lag behind the actual onset of the intestinal injury. Although Thuijls and colleagues [15] demonstrated that urinary I-FABP was a good diagnostic marker for NEC, they concluded that it was not a suitable screening tool for NEC. Rather, Aydemir and colleagues [26] suggested serial measurements of serum I-FABP may be a useful marker for early diagnosis and prediction of severity of NEC.

In our cohort, I-FABP proved to be a sensitive and specific indicator of NEC onset and severity. The trends in I-FABP levels were clearly divergent among the three cohorts of piglets, further supporting its utility as a confirmatory marker of NEC. Piglets with the fulminant phenotype of NEC had a precipitous rise in their serum I-FABP levels, which highly correlated to the extent of disease on pathologic examination, and this presentation was very similar to NEC totalis seen in neonates. The non-fulminant group represents the phenotype that is most commonly seen in human disease. In this subgroup, I-FABP may not only have a role as a diagnostic confirmatory test, but it might also prove useful in monitoring disease progression after intervention efforts to potentially indicate when it is safe to reinitiate enteral feeding. Interestingly, there also appeared to be an exhaustive dose dependent effect in some piglets where their final pre-mortem I-FABP levels were lower than the one prior, suggesting that there was either no further protein to be absorbed into circulation, or severe bowel edema and venous congestion just prior to death prevented any remaining I-FABP from entering into circulation. We also provided compelling evidence supporting the origin of I-FABP in the enterocytes such that rising levels in the plasma are accompanied by lower levels in the intestinal tissue as villi are denuded with progressive necrotic injury. This study was limited by the relatively long 6-h interval between blood sampling, which may have missed the critical window when levels of I-FABP first begin to rise after the initiation of feeds and before the development of clinical symptoms, particularly in the fulminant NEC group. To address this limitation, in future studies we will place special emphasis on the initial 12-h of enteral feeds by decreasing the interval between blood draws to more precisely identify the temporal changes in A-NIRS and pI-FABP levels.

### Limitations

The pathophysiology of NEC is complex and multi-factorial. Several animal models exist to study this disease each of which has its benefits and limitations. Our large animal piglet model of NEC is robust and results in a phenotype of NEC that closely resembles human disease, particularly the non-fulminant NEC that is more insidious. This is a spontaneous model of NEC where hypoxia is not intentionally induced, however, as noted in our findings is an important contributing factor. Our model is subject to the same limitations of translation to the human condition in that human neonates are not permitted to remain hypoxic for any prolonged duration. Although our aim during this study was to observe and examine the A-NIRS and I-FABP changes without intervening. We have since conducted a follow-up study where hypoxic piglets were provided with supplemental oxygen to eliminate hypoxia as a confounding variable to more closely model the human condition.

## Conclusion

In conclusion, this study provides evidence that NIRS is a real-time, non-invasive tool that can serve as a predictive diagnostic modality for necrotizing enterocolitis. We demonstrated that continuously low A-NIRS values and increased A-NIRS variability during initial feeds are highly predictive of NEC in premature piglets and confirmed by rising I-FABP levels in plasma. Furthermore, the study provides insight into the temporal relationships between the development and progression of intestinal tissue injury and changes in both NIRS values and serum I-FABP levels. These modalities used together may help identify neonates with NEC prior to clinical manifestations of the disease. We anticipate these findings will lead to a more focused validation and treatment study in neonates by defining the critical time point where changes in A-NIRS values and increases in pI-FABP best identify the onset of NEC. This information will be essential in translation to clinical practice as it will help guide the appropriate timing of blood draws for pI-FABP in preterm neonates in whom frequent blood sampling is not feasible. Ultimately, we hope to develop A-NIRS as a screening tool for NEC with pI-FABP as a confirmatory test, akin to how ECG and serum cardiac enzyme studies are used for evaluation of cardiac ischemia.

## Supporting Information

S1 Guidelines checklist The ARRIVE (Animal Research: Reporting In Vivo Experiments) Guidelines Checklist. (PDF)
